# Prevalence and cardiovascular phenotypes of dextrocardia and situs inversus among 277,396 adults: longitudinal evidence of amplified age-related blood pressure progression

**DOI:** 10.1186/s13023-026-04449-5

**Published:** 2026-07-07

**Authors:** Dehao Zhang, Mei Lin, Xiaona Wang, Richard Liang, Michelle A. Williams, Yi Ning

**Affiliations:** 1 Key Laboratory of Tropical Translational Medicine of Ministryof Education, School of Public Health, Hainan Academy of Medical Sciences, Hainan Medical University, No. 3 Xueyuan Road, Longhua District, Haikou City, 571199 Hainan Province China; 2 The First School of Clinical Medicine, (The First Affiliated Hospital), Hainan Medical University, Haikou, China; 3 Department of General Practice, School of Clinical Medicine, (Affiliated Hainan Hospital/Hainan General Hospital), Hainan Medical University, Haikou, China; 4 Beijing MJ Healthcare Center, Beijing, China; 5 School of Medicine, Stanford University, Stanford, CA USA; 6 Harvard T.H. Chan School of Public Health, Harvard University, Boston, MA USA; 7 School of Clinical Medicine, (Affiliated Hainan Hospital, Hainan General Hospital), Hainan Medical University, Haikou, China; 8 Shanghai Sci-Tech Inno Center for Infection & Immunity, Shanghai, China

**Keywords:** Dextrocardia, Situs inversus, Prevalence, Nonclinical cohort, Adults, Longitudinal study, Rare diseases, Laterality defects

## Abstract

**Background:**

Dextrocardia and situs inversus are rare congenital malformations. Although associated anomalies are well recognized, their prevalence in unselected adult populations and their cardiovascular phenotypes, particularly blood pressure in nonclinical settings, remain poorly characterized. This study aimed to estimate their prevalence, blood pressure profiles, and longitudinal changes in a large nonclinical cohort.

**Methods:**

In this longitudinal observational study, we reviewed records from the Beijing MJ health check-up cohort (2003–2025). Dextrocardia and situs inversus were confirmed via electrocardiography and imaging. Linear regression models assessed blood pressure differences, adjusting for age, sex, body mass index, glucolipid metabolism, renal function, and lifestyle factors. Linear mixed-effects models evaluated long-term blood pressure changes.

**Results:**

Among 277,396 adults, 43 had dextrocardia, 38 had situs inversus, and 29 had both. The prevalence (95% CI) was 1.6 (1.1–2.0) per 10,000 for dextrocardia and 1.4 (0.9–1.8) per 10,000 for situs inversus. In fully adjusted models, dextrocardia was associated with a 5.3 mmHg higher SBP (95% CI: 1.3–9.2) and a 4.5 mmHg higher DBP (1.7–7.4). Situs inversus was associated with a 7.0 mmHg higher SBP (2.8–11.2) and a 3.3 mmHg higher DBP (0.3–6.3). Longitudinally, each 10-year increase in age was associated with an additional 5.9 mmHg rise in SBP in dextrocardia (1.0–10.7; P for interaction = 0.02) and 5.5 mmHg in situs inversus (0.5–10.6; P for interaction = 0.03) compared with controls.

**Conclusion:**

In this large nonclinical cohort, dextrocardia and situs inversus were associated with higher blood pressure and a steeper age-related increase in SBP. Further studies are needed to confirm these findings.

**Supplementary Information:**

The online version contains supplementary material available at https://doi.org/10.1186/s13023-026-04449-5.

## Introduction

Dextrocardia, defined by a rightward-pointing cardiac apex, and situs inversus, the complete mirror-image reversal of thoracic and abdominal viscera, are rare laterality disorders that may occur in isolation or in combination, such as situs inversus totalis with dextrocardia. Globally, the prevalence of dextrocardia is estimated at 1 in 10,000 to 1 in 12,000 live births [1, 2], while situs inversus ranges from approximately 1 in 6,500 to 1 in 25,000 individuals [3]. Although these anomalies are frequently identified in infancy or childhood through symptoms or imaging, asymptomatic cases often remain undiagnosed into adulthood, especially in the absence of routine screening in nonclinical populations.

These conditions are associated with congenital heart defects, with risk ranging from low (3–5%) in situs inversus with dextrocardia [4, 5] to high (up to 95%) in isolated dextrocardia [6], arising from embryological disruptions such as ciliary dysfunction or mutations in *ZIC3* and *NODAL* [7, 8]. Emerging evidence suggests broader cardiovascular implications, including altered hemodynamics and hypertension in adults [9–13]. However, population-based estimates of prevalence and long-term phenotypes remain limited, as most studies have focused on pediatric or symptomatic cohorts, introducing selection bias and lacking longitudinal data from asymptomatic adults. Nonclinical cohorts are therefore essential for defining the unbiased natural history of these conditions.

This study addresses these gaps in a large longitudinal nonclinical cohort by (1) estimating the prevalence of dextrocardia and situs inversus, (2) characterizing blood pressure profiles, and (3) evaluating longitudinal blood pressure changes, including accelerated age-related progression in participants with dextrocardia or situs inversus compared with those without. Findings from this asymptomatic population should help clarify the cardiovascular implications of these conditions and inform screening and management strategies for affected individuals.

## Methods

### Study population

This cohort study used data from the MJ Health Check-up Cohort [14, 15], an open cohort of nonclinical adults undergoing annual health examinations at MJ Healthcare in Beijing, China (December 2003 to May 2025). Data included laboratory results, imaging, lifestyle factors, medical history, and socioeconomic information obtained through clinical assessments and questionnaires. (1) For prevalence estimation, we used the most recent available records and calculated age as of 2020 to align with the Chinese census [16, 17], excluding participants younger than 20 years or older than 83 years (based on Beijing’s life expectancy of 82.4 years [18]). (2) For blood pressure analyses, we excluded pregnant women, individuals younger than 18 years, steroid hormone users, and records with missing laboratory values or extreme outliers (defined as values below the 0.025th or above the 99.925th percentile). (3) Longitudinal analyses applied the same criteria but included only participants with repeated blood pressure measurements.

The study protocol was approved by the Institutional Review Board of Hainan Medical University (Approval ID: HYLL-2023-449). Informed consent was waived because the study used anonymized retrospective data.

### Assessment of dextrocardia and situs inversus

Dextrocardia was diagnosed using findings from chest X-ray, chest computed tomography (CT), or electrocardiography (ECG). Although not all participants underwent all three modalities at every visit, most received both chest X-ray and ECG during the same examination. Situs inversus was confirmed primarily by abdominal ultrasound, which was performed in nearly all participants at each health check-up. In a small subset of participants, it was also identified on chest X-ray, abdominal X-ray, or upper gastrointestinal barium contrast studies. All diagnoses were independently reviewed by two experienced physicians, with discrepancies resolved by a senior physician to reach consensus.

### Blood pressure measurement and blood sample collection procedures

Blood pressure measurement and blood sample collection were integral parts of the health check-ups and were performed by trained nurses according to standardized protocols. All examinations were conducted in the morning after an overnight fast. Blood samples were collected and analyzed in a central laboratory using fully automated equipment.

For blood pressure assessment, participants rested quietly for at least 5 min before upper-arm blood pressure was measured in the seated position. Blood pressure was measured twice at 30- to 60-second intervals. If the difference was large, a third measurement was obtained, and the average of the two or three readings was recorded.

### Assessment of covariates

Covariates included body mass index (body mass index [BMI], calculated as weight in kilograms divided by height in meters squared), pulse rate and pulse pressure, fasting blood glucose (FBG), lipid profiles (high-density lipoprotein cholesterol [HDL], low-density lipoprotein cholesterol [LDL], triglycerides [TG], and total cholesterol [TC]), estimated glomerular filtration rate (eGFR) using the CKD-EPI 2021 equation [19], and questionnaire-derived data on medical and family history, medications, lifestyle (smoking and drinking), education, left-handedness, and twin status.

Health conditions were defined using a combination of measured values, self-reports, or medication use: hypertension was defined as systolic blood pressure (SBP) ≥ 140 mmHg or diastolic blood pressure (DBP) ≥ 90 mmHg, self-reported diagnosis, or use of antihypertensive medication; diabetes as FBG ≥ 7.0 mmol/L, self-reported diagnosis, or use of antidiabetic medication; and dyslipidemia as abnormal lipid levels (TG ≥ 2.3 mmol/L or TC ≥ 6.2 mmol/L or HDL < 1.0 mmol/L or LDL ≥ 4.1 mmol/L), self-reported diagnosis, or use of lipid-lowering medication.

### Statistical analyses

Age in 2020 was calculated as 2020 minus birth year. Prevalence was reported as both crude and weighted estimates using the 2020 Chinese census data, stratified by sex and 5-year age groups. Prevalence estimates and 95% confidence intervals (CIs) were calculated using Taylor series linearization to account for the complex survey design. Sex differences in unweighted and weighted prevalence were assessed using Fisher’s exact test and the Rao-Scott chi-square test, respectively.

Participants in the blood pressure difference analysis were categorized into the following groups: those without dextrocardia or situs inversus, overall dextrocardia, overall situs inversus, dextrocardia with situs inversus, isolated dextrocardia, and isolated situs inversus. Data are presented as geometric least-squares means (95% CIs) for continuous variables and numbers (percentages) for categorical variables. Characteristics of participants with dextrocardia or situs inversus were compared with those without either condition. Between-group differences were quantified using standardized mean differences (SMD). For continuous variables, SMD was calculated as the difference in means divided by the pooled standard deviation. For categorical variables, SMD was computed using Cohen’s h, based on the arcsine transformation of proportions. Age-related blood pressure trends were visualized using locally estimated scatterplot smoothing (LOESS) and compared with age-matched peers without these conditions.

In the blood pressure differences analyses, we used general linear models to estimate beta coefficients (β) and 95% CI for the association between dextrocardia and situs inversus and blood pressure. The fully adjusted model accounted for sex (male, female), age, BMI, FBG, LDL, eGFR, current antihypertensive medication use (yes, no), family history of hypertension (yes, no), current drinking status (never, former, 1–2 times per week, 3–4 times per week, daily), current smoking status (never, former, < 1 time per day, daily), and educational attainment (junior high or below, high school graduate, bachelor’s degree, postgraduate degree). Missing continuous values were imputed by matched means (same sex, ± 2 years age, ± 1 BMI) (only among participants with dextrocardia or situs inversus). Missing categorical data were handled using the indicator method.

To assess the robustness of our findings, we conducted sensitivity analyses: (1) excluding dextrocardia cases not confirmed by imaging (X-ray or CT) to verify diagnostic accuracy; (2) excluding those with missing continuous variable values to evaluate imputation effects; (3) excluding those with missing data on smoking, drinking, or education to test the impact of incomplete covariate information; (4) substituting diabetes and dyslipidemia status for current FBG and LDL level to assess potential unmeasured confounders underlying these disease states, beyond current metabolic control levels. Subgroup analyses were conducted according to sex (male, female), age (≤ 40, > 40 years), BMI (< 24, ≥ 24 kg/m^2^), combine with situs inversus or dextrocardia (yes, no), and family history of hypertension (yes, no).

We used linear mixed-effects models to analyze the characteristics of blood pressure changes with age in participants with dextrocardia and situs inversus. Only participants with repeated measurements of SBP, DBP, and pulse pressure at multiple time points were included. In the models, we incorporated random intercepts and random slopes to capture individual variability in baseline blood pressure and its trajectory over time. All statistical analyses were conducted using R software (Version 4.4.2). All tests were two-sided, and statistical significance was defined as *P* < 0.05.

## Results

### Prevalence of dextrocardia and situs inversus

Among 277,396 adults aged ≥ 20 years (Fig. S1), 43 had dextrocardia, 38 had situs inversus, and 29 had both. The crude prevalence was 1.6 (95% CI: 1.1–2.0) per 10,000 for dextrocardia, 1.4 (95% CI: 0.9–1.8) per 10,000 for situs inversus, and 1.0 (95% CI: 0.7–1.4) per 10,000 for both conditions (Table 1). Weighted estimates based on Beijing and national census data were similar to the crude rates, with no significant sex differences. Point estimates were consistently higher in females than in males for all three conditions, although no statistically significant sex differences were observed (all P values > 0.05).

**Table 1:** Estimated prevalence of dextrocardia and situs inversus by sex among population aged over 20 years in Beijing and nationwide, China, 2020

	Number of participants	Crude		City’s census weighted		Nation’s census weighted	
	with or without condition	prevalence (95% CI)		prevalence (95% CI)		prevalence (95% CI)	
Dextrocardia							
Overall	43/277,353	1.6 (1.1-2.0)		1.5 (1.0–2.0)		1.6 (1.0-2.1)	
Male	20/143,215	1.4 (0.8-2.0)		1.3 (0.7-2.0)		1.3 (0.7-2.0)	
Female	23/134,138	1.7 (1.0-2.4)		1.7 (0.9–2.5)		1.8 (1.0-2.6)	
P value	-	0.5		0.4		0.4	
Situs inversus							
Overall	38/277,358	1.4 (0.9–1.8)		1.4 (0.9–1.9)		1.4 (0.9–1.9)	
Male	16/143,219	1.1 (0.6–1.7)		1.2 (0.5–1.8)		1.1 (0.5–1.7)	
Female	22/134,139	1.6 (1.0-2.3)		1.6 (0.9–2.4)		1.7 (0.9–2.5)	
P value	-	0.3		0.4		0.3	
Dextrocardia with situs inversus							
Overall	29/277,367	1.0 (0.7–1.4)		1.0 (0.6–1.4)		1.0 (0.6–1.4)	
Male	13/143,222	0.9 (0.4–1.4)		0.9 (0.4–1.4)		0.9 (0.4–1.4)	
Female	16/134,145	1.2 (0.6–1.8)		1.1 (0.5–1.8)		1.2 (0.5–1.8)	
P value	-	0.6		0.6		0.5	

All included participants were aged ≥ 20 years in 2020

Crude and weighted Prevalence was expressed as the number of dextrocardia individuals per 10,000 population

According to the 2020 China National Census, Beijing had 18,668,031 residents aged ≥ 20 years (males and females accounted for 50.9% and 49.1%, respectively), while nationally there were 1,083,710,646 adults ≥ 20 years (males and females accounted for 50.5% and 49.5%, respectively)

The P values for sex difference in crude prevalence was calculated using Fisher’s exact test. For weighted prevalence comparisons between sex, P-values were derived from Rao-Scott chi-square tests

### Characteristics of participants with dextrocardia and situs inversus

A total of 227,834 participants without dextrocardia or situs inversus, 43 with dextrocardia (29 with situs inversus and 14 isolated), and 38 with situs inversus (9 isolated) were included (Fig. S2). Demographic and clinical characteristics are summarized in Table 2. Participants with dextrocardia or situs inversus were shorter than controls (163–165 vs. 167 cm; SMD ≥ 0.2). The proportion of males was lower in the situs inversus group (42% overall, 33% in isolated cases) than in controls (54%; SMD ≥ 0.2). Participants with isolated dextrocardia were older (48 vs. 42 years; SMD ≥ 0.2).

**Table 2:** Demographic and clinical features of participants with dextrocardia and situs inversus

Characteristics	Without dextrocardia or situs inversus	Overall dextrocardia	Overall situs inversus	Dextrocardia with situs inversus	Isolated dextrocardia	Isolated situs inversus
Number of participants	227,834	43	38	29	14	9
Age, years	42 (42–42)	42 (38–46)	41 (37–44)	39 (35–44)	48 (41–54)^*^	44 (36–52)
Males, n (%)	122,187 (54)	20 (47)	16 (42)^*^	13 (45)	7 (50)	3 (33)^*^
Body height, cm	167 (167–167)	165 (162–167)^*^	165 (162–168)^*^	165 (162–168)^*^	163 (159–168)^*^	165 (159–170)^*^
Body mass index, kg/m^2^	24 (24–24)	24 (23–25)	24 (22–25)	24 (22–25)	24 (22–26)	24 (21–26)
Systolic blood pressure, mmHg	115 (114–115)	120 (115–125)^*^	121 (116–126)^*^	119 (113–124)^*^	123 (114–131)^*^	130 (119–140)^*^
Diastolic blood pressure, mmHg	70 (70–70)	75 (71–78)^*^	73 (70–77)^*^	73 (69–77)^*^	78 (72–84)^*^	72 (65–80)
Pulse pressure, mmHg	44 (44–44)	45 (42–48)	48 (45–51)^*^	45 (41–49)	45 (39–50)	57 (50–64)^*^
Pulse rate, bpm	66 (66–66)	67 (64–69)	69 (66–72)^*^	67 (64–70)	66 (61–71)	75 (69–80)^*^
Fasting blood glucose, mmol/L	5.6 (5.6–5.6)	5.6 (5.3–5.9)	5.7 (5.4-6.0)	5.6 (5.3-6.0)	5.6 (5.1–6.1)	5.8 (5.2–6.4)^*^
Total cholesterol, mmol/L	4.7 (4.7–4.7)	4.6 (4.3–4.9)	4.5 (4.2–4.8)^*^	4.5 (4.2–4.8)^*^	4.8 (4.4–5.3)	4.6 (4.1–5.2)
Triglycerides, mmol/L	1.3 (1.3–1.3)	1.3 (1.0-1.6)	1.2 (0.9–1.5)	1.2 (0.9–1.5)	1.5 (1.0–2.0)	1.3 (0.7–1.8)
Low-density lipoprotein cholesterol, mmol/L	2.9 (2.9–2.9)	2.8 (2.6–3.1)	2.8 (2.5–3.1)	2.7 (2.4-3.0)^*^	3.1 (2.6–3.5)^*^	3.0 (2.4–3.7)^*^
High-density lipoprotein cholesterol, mmol/L	1.4 (1.4–1.4)	1.4 (1.3–1.5)	1.4 (1.2–1.5)	1.4 (1.2–1.5)	1.4 (1.2–1.6)	1.4 (1.2–1.7)
Estimated glomerular filtration rate, mL/min/1.73 m^2^	107 (107–107)	110 (105–114)	109 (105–114)	111 (105–116)^*^	108 (100–115)	106 (97–115)
Hypertension, n (%)	36,557 (16)	11 (25.6)^*^	10 (26)^*^	5 (17)	6 (43)^*^	5 (56)^*^
Diabetes, n (%)	61,872 (27)	14 (32.6)	16 (42)^*^	12 (41)^*^	2 (14)^*^	4 (44)^*^
Dyslipidemia, n (%)	109,358 (48)	17 (39.5)	13 (34)^*^	9 (31)^*^	8 (57)	4 (44)
Current antihypertensive medication use, n (%)	20,398 (9)	4 (9)	6 (15.8)^*^	3 (10)	1 (7)	3 (33)^*^
Family history of hypertension, n (%)	88,552 (39)	21 (49)^*^	16 (42)	14 (48)	7 (50)^*^	2 (22)^*^
Perceived health status worse than peers, n (%)	15,319 (14)	3 (15.8)	2 (15)	1 (9)	2 (25)^*^	1 (50)^*^
Twins, n (%)	4,427 (3)	0 (0)^*^	0 (0)^*^	0 (0)^*^	0 (0)^*^	0 (0)^*^
Left-handed habits, n (%)	9,704 (7)	1 (3)	1 (5)	1 (5.6)	0 (0)^*^	0 (0)^*^
Current drinking, n (%)	46,872 (23)	7 (17.5)	4 (11)	4 (14)^*^	3 (25)	0 (0)^*^
Current smoking, n (%)	50,579 (24)	11 (27)	9 (25.7)	7 (25)	4 (31)	2 (29)
College education, n (%)	145,790 (88)	23 (68)^*^	22 (79)^*^	18 (78)^*^	5 (46)^*^	4 (80)^*^

Data are presented as geometric least-squares means (95% confidence intervals) for continuous variables and as numbers (percentages) for categorical variables

* indicates comparison with the participants without dextrocardia or situs inversus, shows the standardized mean difference equal to or more than 0.2

Missing data were not included in the estimation and calculation of geometric least-squares means (95% confidence intervals) and percentages

Missing data among participants without dextrocardia or situs inversus, the variables of perceived health status (*n* = 120,079), twins (*n* = 86,017), left-handed habits (*n* = 90,988), drinking status (*n* = 27,245), smoking status (*n* = 17,277), and educational attainment (*n* = 61,710) with missing data; among participants with dextrocardia (overall), the variables of low-density lipoprotein cholesterol (*n* = 6), high-density lipoprotein cholesterol (*n* = 6), perceived health status (*n* = 24), twins (*n* = 14), left-handed habits (*n* = 13), drinking status (*n* = 3), smoking status (*n* = 2), and educational attainment (*n* = 9) with missing data; among participants with isolated dextrocardia, the variables of perceived health status (*n* = 6), twins (*n* = 3), left-handed habits (*n* = 2), drinking status (*n* = 2), smoking status (*n* = 1), and educational attainment (*n* = 3) with missing data; among participants with dextrocardia and situs inversus, the variables of low-density lipoprotein cholesterol (*n* = 6), high-density lipoprotein cholesterol (*n* = 6), perceived health status (*n* = 18), twins (*n* = 11), left-handed habits (*n* = 11), drinking status (*n* = 1), smoking status (*n* = 1), and educational attainment (*n* = 6) with missing data; among participants with situs inversus (overall), the variables of low-density lipoprotein cholesterol (*n* = 8), high-density lipoprotein cholesterol (*n* = 8), perceived health status (*n* = 25), twins (*n* = 15), left-handed habits (*n* = 18), drinking status (*n* = 3), smoking status (*n* = 3), and educational attainment (*n* = 10) with missing data; among participants with isolated situs inversus, the variables of low-density lipoprotein cholesterol (*n* = 2), high-density lipoprotein cholesterol (*n* = 2), perceived health status (*n* = 7), twins (*n* = 4), left-handed habits (*n* = 7), drinking status (*n* = 2), smoking status (*n* = 2), and educational attainment (*n* = 4)

### Cardiovascular risk factors

**Fig. 1:**
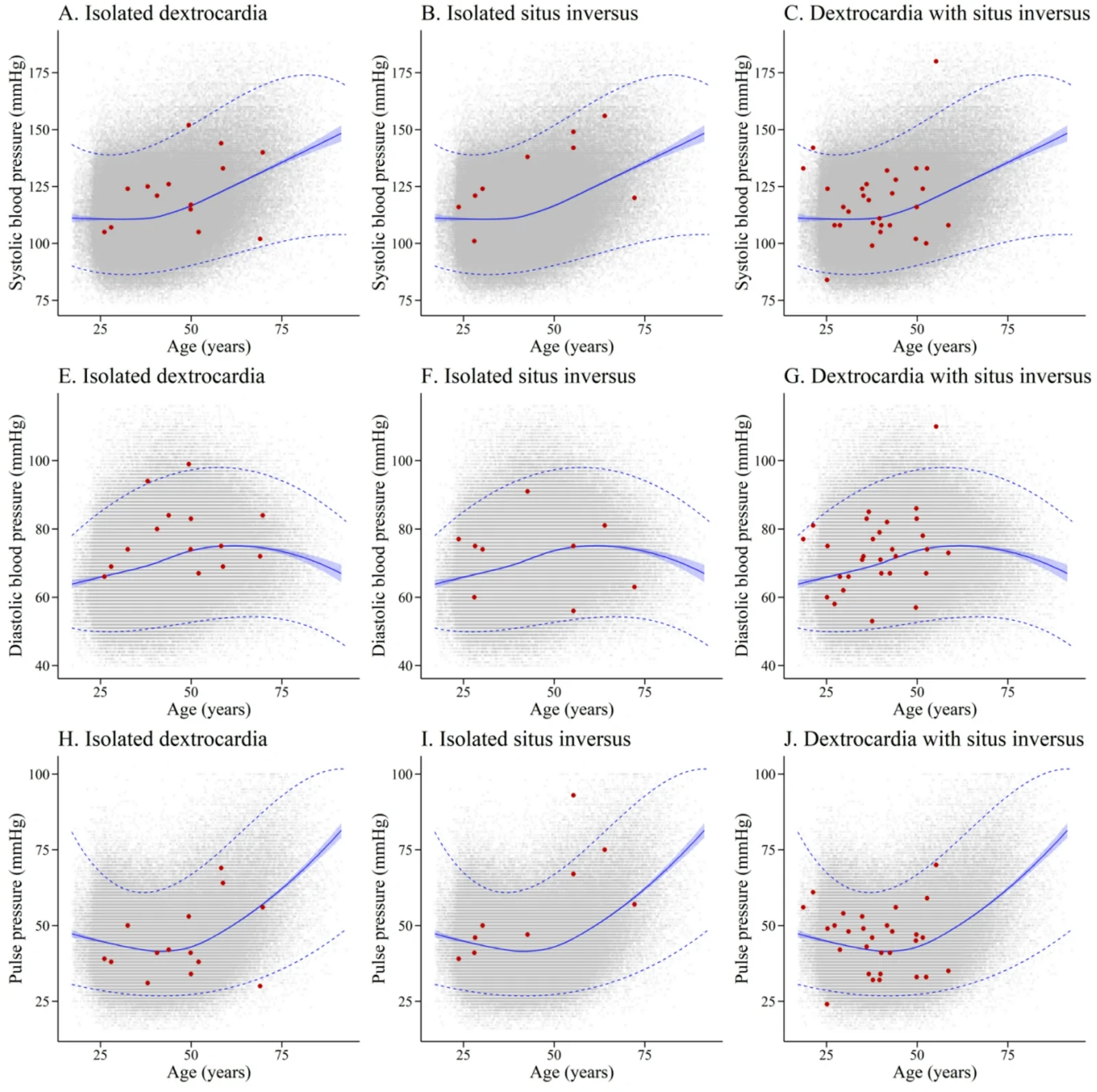
Characteristics of blood pressure of participants with dextrocardia or situs inversus by age. The red dots represent blood pressure measurements from participants with dextrocardia or situs inversus across different ages; the gray dots represent blood pressure measurements from 227,834 participants without dextrocardia or situs inversus. The solid blue line (10% random sample of 227,834 participants), blue shaded area (10% random sample of 227,834 participants), and blue dashed lines (227,834 participants) indicate the LOESS-smoothed mean, standard error of the mean, and 95th percentile range (upper and lower) for participants without dextrocardia or situs inversus at different ages, respectively. The LOESS-smoothed mean was calculated using local polynomial regression model, while the 2.5th and 97.5th percentiles where estimated by quantile regression model. Due to the substantial computational memory requirements for calculating standard errors in the LOESS model, a 10% random subsample of participants without dextrocardia or situs inversus was used to estimate the mean trend and its associated standard error. The loess-smoothed mean curve estimated using the full sample (227,834 participants without sutis inversus, Fig. S4) was nearly identical to that obtained from the 10% random subsample

Participants with dextrocardia or situs inversus exhibited higher blood pressure than controls, with SBP 4–15 mmHg higher and DBP 3–8 mmHg higher (all SMD ≥ 0.2). Age-stratified pattern (Fig. 1, based on a 10% random sample of the 227,834 controls; full results in Fig. S4) showed that SBP and DBP in affected participants were consistently higher than in age-matched controls across ages 25–75 years, although they remained within the 95th percentile of the control distribution. Hypertension prevalence was substantially higher in isolated dextrocardia (43% vs. 16%) and isolated situs inversus (56% vs. 16%), whereas family history of hypertension was more common in participants with dextrocardia (49–50% vs. 39%; all SMD ≥ 0.2).

### Metabolic profiles and kidney function

Fasting blood glucose levels were comparable across groups, with no clinically meaningful differences. However, diabetes prevalence was substantially higher among participants with situs inversus (both overall and isolated) and those with dextrocardia combined with situs inversus (41–44% vs. 27% in controls; SMD ≥ 0.2). In contrast, dyslipidemia prevalence was lower in the situs inversus groups (31–34% vs. 48% in controls; SMD ≥ 0.2). Other lipid parameters and eGFR showed only minor differences of uncertain clinical relevance (see Table 2 for details).

### Association between dextrocardia and situs inversus and blood pressure

In the fully adjusted model (multivariable-adjusted model 2, Table 3), dextrocardia was associated with 5.3 mmHg higher SBP (β: 5.3 mmHg, 95% CI: 1.3–9.2) and 4.5 mmHg higher DBP (4.5 mmHg, 1.7–7.4), with no significant difference in pulse pressure (0.7 mmHg, − 2.3 to 3.7). Situs inversus was associated with 7.0 mmHg higher SBP (7.0 mmHg, 2.8–11.2), 3.3 mmHg higher DBP (3.3 mmHg, 0.3–6.3), and 3.7 mmHg higher pulse pressure (3.7 mmHg, 0.5–6.9).

**Table 3:** Multivariate-adjusted beta coefficients and 95% confidence intervals for blood pressure differences by dextrocardia and situs inversus status

	Crude model		Mv-adj model 1		Mv-adj model 2	
	β, mmHg	(95% CI)	β, mmHg	(95% CI)	β, mmHg	(95% CI)
Dextrocardia						
Systolic blood pressure	5.3^*^	(0.6, 10.1)	5.7^*^	(1.7, 9.7)	5.3^*^	(1.3, 9.2)
Diastolic blood pressure	4.4^*^	(1.1, 7.7)	4.7^*^	(1.9, 7.6)	4.5^*^	(1.7, 7.4)
Pulse pressure	0.9	(-2.3, 4.1)	0.9	(-2.1, 3.9)	0.7	(-2.3, 3.7)
Situs inversus						
Systolic blood pressure	6.7^*^	(1.6, 11.7)	7.2^*^	(2.9, 11.4)	7.0^*^	(2.8, 11.2)
Diastolic blood pressure	2.7	(-0.8, 6.3)	3.3^*^	(0.2, 6.3)	3.3^*^	(0.3, 6.3)
Pulse pressure	3.9^*^	(0.5, 7.3)	3.9^*^	(0.7, 7.1)	3.7^*^	(0.5, 6.9)

Crude model is unadjusted. Multivariate adjusted model 1 adjusted for sex, age, body mass index, fasting blood glucose, low-density lipoprotein cholesterol (including imputation of missing values for 6 individuals with dextrocardia), estimated glomerular filtration rate, current antihypertensive medication use, and family history of hypertension. Multivariate adjusted model 2 further plus current drinking status, current smoking status, and educational attainment

Reference groups were participants without dextrocardia or situs inversus for their respective comparisons

^*^ indicates statistical significance at P value less than 0.05

Abbreviation: Mv-adj model, multivariate adjusted model; β, beta coefficients; CI, confidence interval

### Sensitivity analyses

Results of the sensitivity analyses were largely consistent with the main findings in Table 3 (Table 4). For dextrocardia, the β for SBP ranged from a decrease of 18.9% (to 4.3 mmHg) in imaging-confirmed cases (*n* = 34) to an increase of 20.8% (to 6.4 mmHg) when excluding participants with missing data on drinking, smoking, and education (*n* = 33). The β for DBP varied modestly, from a 6.7% decrease (to 4.2 mmHg, *n* = 34) to an 8.9% increase (to 4.9 mmHg, *n* = 37 and 33).

**Table 4:** Sensitive analyses for blood pressure differences by dextrocardia and situs inversus status

	Systolic blood pressure		Diastolic blood pressure		Pulse pressure	
	Mv-adj β, mmHg	(95% CI)	Mv-adj β, mmHg	(95% CI)	Mv-adj β, mmHg	(95% CI)
Excluding non-imaging-confirmed						
Dextrocardia (*n* = 34)	4.3	(-0.1, 8.8)	4.2^*^	(1.0, 7.4)	0.2	(-3.2, 3.5)
Excluding missing value on low-density lipoprotein						
Cholesterol						
Dextrocardia (*n* = 37)	5.2^*^	(1.0, 9.5)	4.9^*^	(1.9, 8.0)	0.3	(-2.9, 3.5)
Situs inversus (*n* = 30)	7.0^*^	(2.3, 11.7)	3.2	(-0.2, 6.6)	3.8^*^	(0.2, 7.4)
Excluding missing value on drinking, smoking status						
and educational attainment						
Dextrocardia (*n* = 33)	6.4^*^	(1.9, 10.9)	4.9^*^	(1.7, 8.2)	1.5	(-1.9, 4.9)
Situs inversus (*n* = 27)	7.9^*^	(2.9, 12.9)	5.2^*^	(1.6, 8.8)	2.7	(-1.1, 6.5)
Replacing covariates with disease status in models						
Dextrocardia (*n* = 43)	5.4^*^	(1.4, 9.3)	4.7^*^	(1.8, 7.5)	0.7	(-2.3, 3.7)
Situs inversus (*n* = 38)	7.4^*^	(3.2, 11.7)	3.7^*^	(0.7, 6.7)	3.8^*^	(0.6, 6.9)

Multivariate adjusted model adjusted for sex, age, body mass index, fasting blood glucose, low-density lipoprotein cholesterol (including imputation of missing values for 6 individuals with dextrocardia), estimated glomerular filtration rate, current antihypertensive medication use, family history of hypertension, current drinking status, current smoking status, and educational attainment

Reference groups were participants without dextrocardia or situs inversus for their respective comparisons

^*^ indicates statistical significance at P value less than 0.05

Abbreviation: Mv-adj model, multivariate adjusted model; β, beta coefficients; CI, confidence interval

For situs inversus, the β for SBP remained stable (maximum change 12.9%, to 7.9 mmHg, *n* = 27). The β for DBP showed greater variation, decreasing by 3.0% (to 3.2 mmHg, *n* = 30) after excluding missing LDL values and increasing by 57.6% (to 5.2 mmHg, *n* = 27) when participants with missing data on drinking, smoking, and education were excluded. Further details are presented in Table 4.

### Subgroup analyses

**Fig. 2:**
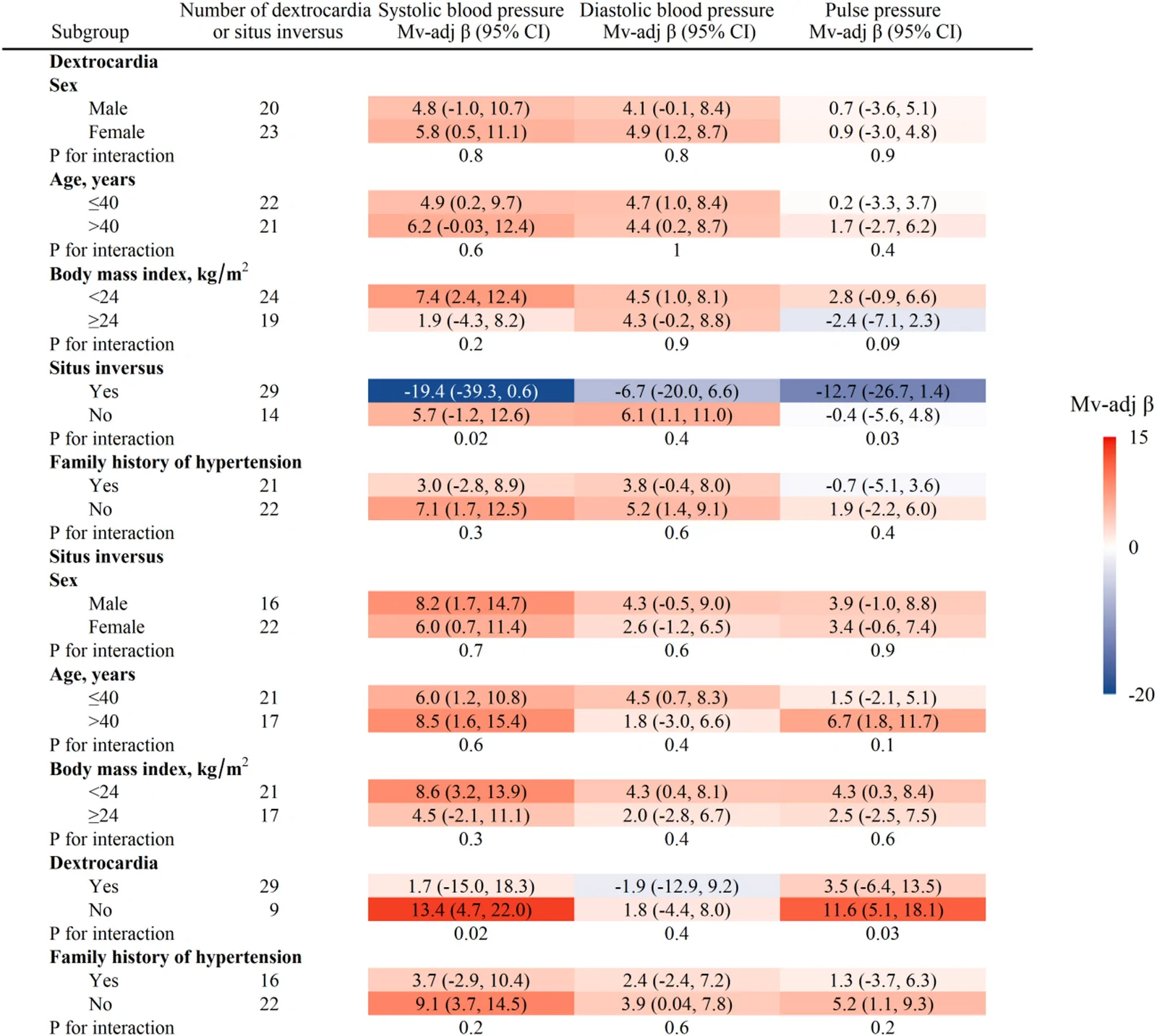
Subgroup analyses of blood pressure characteristics in participants with dextrocardia or situs inversus across sex, age, body mass index, and family history of hypertension. Multivariate adjusted model adjusted for sex, age, body mass index, fasting blood glucose, low-density lipoprotein cholesterol (including imputation of missing values for 6 individuals with dextrocardia), estimated glomerular filtration rate, current antihypertensive medication use, family history of hypertension, current drinking status, current smoking status, and educational attainment. Reference groups were participants without dextrocardia or situs inversus for their respective comparisons. Abbreviation: Mv-adj model, multivariate adjusted model; β, beta coefficients; CI, confidence interval

Figure 2 shows the results of subgroup analyses for blood pressure differences associated with dextrocardia or situs inversus. The blood pressure differences were consistent across subgroups defined by sex, age, BMI, and family history of hypertension (all P for interaction > 0.05). However, a significant interaction was observed with situs inversus: the association with SBP was more negative among those with situs inversus (β: −19.4 mmHg, 95% CI: −39.3 to 0.6) than among those without (5.7 mmHg, − 1.2 to 12.6; P for interaction = 0.02). Similarly, the association with SBP was stronger among participants without dextrocardia (13.4 mmHg, 4.7–22.0) than among those with dextrocardia (1.7 mmHg, − 15.0 to 18.3; P for interaction = 0.02). Pulse pressure showed similar patterns to SBP, with significant interactions for both dextrocardia and situs inversus (both P for interaction = 0.03). Given the small sample sizes in the isolated subgroups (*n* = 9–14), these exploratory subgroup interactions should be interpreted with great caution and regarded as hypothesis-generating.

### Blood pressure differences in isolated dextrocardia, isolated situs inversus, and both conditions

Given the potential differences observed in the subgroup analyses (Fig. 2), we conducted further exploratory analyses to examine blood pressure differences according to specific congenital conditions. Compared with participants without either condition, isolated dextrocardia (*n* = 14) was associated with 5.7 mmHg higher SBP (95% CI: −1.2 to 12.6) and dextrocardia with situs inversus (*n* = 29) with 5.1 mmHg higher SBP (0.2 to 9.9). DBP was significantly higher in both groups (6.1 mmHg [1.1 to 11.0] for isolated dextrocardia and 3.8 mmHg [0.3 to 7.2] for dextrocardia with situs inversus). Isolated situs inversus (*n* = 9) was associated with 13.4 mmHg higher SBP (95% CI: 4.7 to 22.0). Pulse pressure was also higher (11.6 mmHg, 5.1 to 18.1), whereas DBP was not significantly different (1.8 mmHg, − 4.4 to 8.0; Table 5). Owing to the limited sample sizes in the isolated subgroups, these findings are considered preliminary.

**Table 5:** Multivariate-adjusted beta coefficients and 95% confidence intervals for blood pressure differences among participants with isolated dextrocardia, isolated situs inversus, and dextrocardia with situs inversus, compared to those without either condition

	Number of participants withdextrocardia	Systolic blood pressure		Diastolic blood pressure		Pulse pressure	
	or/and situs inversus	Mv-adj β, mmHg	(95% CI)	Mv-adj β, mmHg	(95% CI)	Mv-adj β, mmHg	(95% CI)
Isolated dextrocardia	14	5.7	(-1.2, 12.6)	6.1^*^	(1.1, 11.0)	-0.4	(-5.6, 4.8)
Isolated situs inversus	9	13.4^*^	(4.7, 22.0)	1.8	(-4.4, 8.0)	11.6^*^	(5.1, 18.1)
Dextrocardia with situs inversus	29	5.1^*^	(0.2, 9.9)	3.8^*^	(0.3, 7.2)	1.3	(-2.4, 4.9)

Multivariate adjusted model adjusted for sex, age, body mass index, fasting blood glucose, low-density lipoprotein cholesterol (including imputation of missing values for 6 individuals with dextrocardia), estimated glomerular filtration rate, current antihypertensive medication use, family history of hypertension, current drinking status, current smoking status, and educational attainment

Reference group was 227,834 participants without both dextrocardia and situs inversus

^*^ indicates statistical significance at P value less than 0.05

Abbreviation: Mv-adj model, multivariate adjusted model; β, beta coefficients; CI, confidence interval

### Longitudinal blood pressure changes

**Fig. 3:**
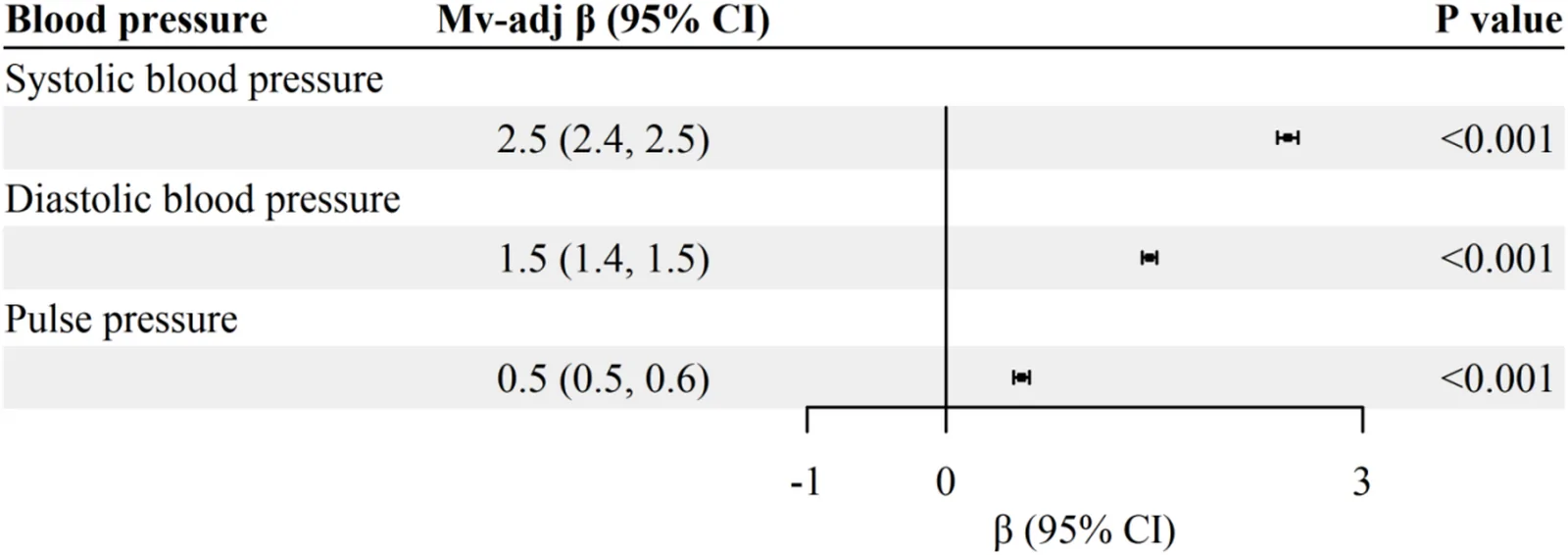
Longitudinal patterns of blood pressure change with aging in 87,659 participants without dextrocardia or situs inversus. Multivariate adjusted model adjusted for sex, age, body mass index, fasting blood glucose, low-density lipoprotein cholesterol, estimated glomerular filtration rate, current antihypertensive medication use, family history of hypertension, current drinking status, current smoking status, and educational attainment. Abbreviation: Mv-adj model, multivariate adjusted model; β, beta coefficients; CI, confidence interval

**Fig. 4:**
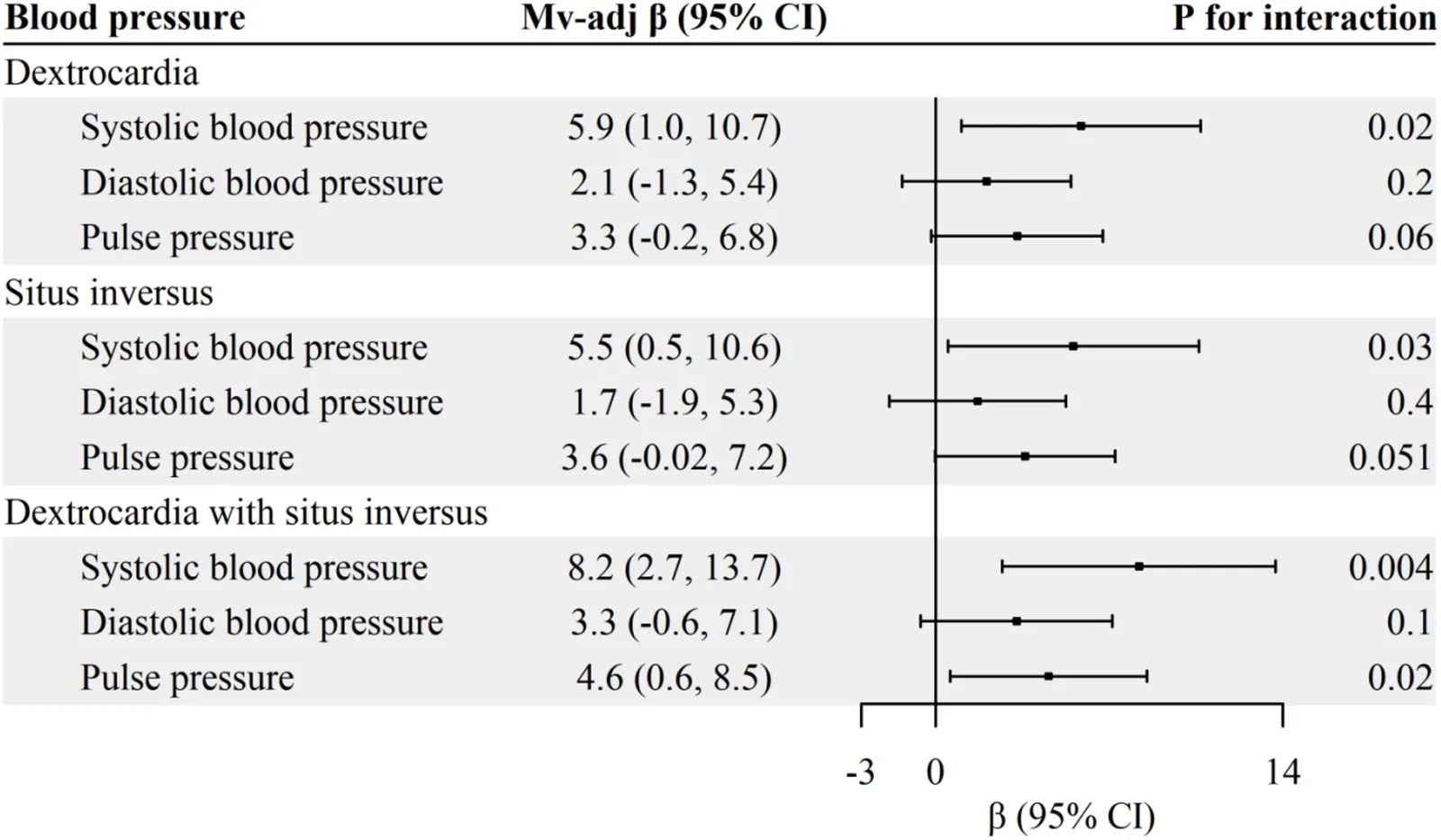
Differential longitudinal blood pressure patterns between participants with dextrocardia or situs inversus and both of these conditions. Dextrocardia, situs inversus, and dextrocardia with situs inversus analyses included 18, 14, and 13 participants, respectively. Multivariate adjusted model adjusted for sex, age, body mass index, fasting blood glucose, low-density lipoprotein cholesterol, estimated glomerular filtration rate, current antihypertensive medication use, family history of hypertension, current drinking status, current smoking status, and educational attainment. Abbreviation: Mv-adj model, multivariate adjusted model; β, beta coefficients; CI, confidence interval

Figure 3 shows age-related blood pressure changes in 87,659 participants without dextrocardia or situs inversus (Fig. S3 shows the participant flow chart; mean follow-up time was 4.4 years). Among participants without dextrocardia or situs inversus, each 10-year increase in age was associated with a 2.5 mmHg rise in SBP (95% CI: 2.4–2.5), a 1.5 mmHg rise in DBP (1.4–1.5), and a 0.5 mmHg rise in pulse pressure (0.5–0.6). Participants with dextrocardia had an additional 5.9 mmHg rise in SBP per 10-year age increase (95% CI: 1.0–10.7; P for interaction = 0.02), those with situs inversus had an additional 5.5 mmHg rise (0.5–10.6; P for interaction = 0.03), and those with both conditions had an additional 8.2 mmHg rise (2.7–13.7; P for interaction = 0.004). Differences in DBP and pulse pressure were also observed, although their statistical significance varied (Fig. 4).

## Discussion

This study provides a detailed examination of dextrocardia and situs inversus in a large nonclinical cohort. The prevalence of dextrocardia was estimated at 1.6 per 10,000, situs inversus at 1.4 per 10,000, and both conditions at 1.0 per 10,000. Participants with these conditions exhibited higher blood pressure, with the largest point estimate observed in those with isolated situs inversus. Longitudinal analyses further showed a steeper age-related increase in systolic blood pressure among participants with dextrocardia, situs inversus, or both conditions compared with those without either condition.

Our prevalence estimates are consistent with those from previous studies in diverse populations, although minor variations may arise from differences in detection methods and cohort characteristics. For example, dextrocardia has been reported at approximately 1 in 10,000 to 12,000 live births (0.8–1.0 per 10,000) [1, 20], and situs inversus ranges from 1 in 6,500 to 25,000 (0.4–1.5 per 10,000) [3, 21], consistent with our findings. Although not statistically significant, our study showed a numerically higher prevalence of these conditions among females, in contrast to prior reports of a male predominance in situs inversus [3]. This finding merits further investigation in larger cohorts.

The higher blood pressure observed in this study adds important new information, as previous evidence on cardiovascular phenotypes in dextrocardia and situs inversus has been largely anecdotal or limited to case reports [9–13]. Prior case reports have documented hypertension in individuals with these conditions, frequently in the setting of Kartagener syndrome (primary ciliary dyskinesia with situs inversus) [9, 22, 23]. However, our study is the first to quantify these associations in a large asymptomatic population after adjustment for metabolic, renal, and lifestyle factors. We also observed a steeper age-related rise in systolic blood pressure observed in our cohort is particularly novel, as no prior longitudinal studies in nonclinical cohorts have addressed this issue.

Mechanistically, these findings may relate to embryological disruptions in left-right asymmetry, often involving ciliary dysfunction or mutations in genes such as *ZIC3* and *NODAL*, as noted in the introduction. Ciliary defects, which are prevalent in situs inversus [24, 25], can impair nitric oxide signaling [26], a pathway that regulates vascular tone and endothelial function [27]. The apparently stronger association observed in participants with isolated situs inversus raises the possibility of distinct physiological effects related to complete visceral mirroring. However, subgroup analyses showed interactions, which should be interpreted with caution due to the small numbers of participants in the isolated dextrocardia and isolated situs inversus groups. These findings should be regarded as hypothesis-generating rather than definitive evidence of effect modification. Beyond primary laterality-related mechanisms, other pathophysiological processes may be relevant, including autonomic nervous system dysregulation, increased electrical repolarization heterogeneity, and subclinical myocardial and vascular remodeling. Case reports have described disturbances in autonomic control in individuals with situs inversus, including arrhythmias, sick sinus syndrome, and ventricular tachyarrhythmias [28]. In addition, diffusion tensor cardiac magnetic resonance imaging has demonstrated deranged myocyte microstructural organization in situs inversus totalis [29].

Strengths of our study include the systematic assessment of dextrocardia and situs inversus in a large nonclinical cohort (*n* = 277,396 adults) with a longitudinal design, which reduces ascertainment bias inherent in hospital-based research. Comprehensive covariate adjustment, sensitivity analyses, and linear mixed-effects models further strengthen the robustness of our conclusions. Nonetheless, several limitations should be acknowledged. Due to the rarity of these conditions, the relevant subgroups, particularly the isolated forms, were small. This led to limited statistical power for interaction tests and wide confidence intervals. Accordingly, given the small numbers in the isolated subgroups, the observed interactions should be interpreted with caution and regarded as hypothesis-generating. Diagnostic reliance on routine imaging may have missed subtle cases, although dual-physician review helped mitigate this. Notably, participants with situs inversus exhibited a substantially higher prevalence of diabetes (41–44% vs. 27% in controls). Although the primary analyses adjusted for fasting blood glucose, and sensitivity analyses using binary diabetes status yielded consistent results, residual confounding from this imbalance cannot be entirely excluded. We lacked genetic or ciliary function data, precluding mechanistic stratification (e.g., primary ciliary dyskinesia versus non-primary ciliary dyskinesia). The predominantly Chinese cohort may limit generalizability to other ethnic groups; residual confounding from unmeasured factors also cannot be fully excluded. Future studies in larger, multi-ethnic cohorts are needed to confirm these associations and evaluate potential cardiovascular health risks in these rare conditions using non-invasive approaches, such as machine learning-enhanced ECG methods, building on recent methodological advances [30].

## Conclusion

In conclusion, this study demonstrates that dextrocardia and situs inversus are associated with higher blood pressure and a steeper age-related increase in systolic blood pressure in adults in this nonclinical cohort. These associations underscore the importance of enhanced cardiovascular surveillance in affected individuals, although causality cannot be inferred from observational data alone.

## Supplementary Information

Below is the link to the electronic supplementary material.

Supplementary Material 1

## Data Availability

The datasets generated and/or analyzed during the current study are not publicly available due to participant confidentiality and ethical restrictions but are available from the corresponding author upon reasonable request.

## Abbreviations

SBP: Systolic blood pressure
DBP: Diastolic blood pressure
CI: Confidence interval
BMI: Body mass index
FBG: Fasting blood glucose
HDL: High-density lipoprotein cholesterol
LDL: Low-density lipoprotein cholesterol
TG: Triglycerides
TC: Total cholesterol
eGFR: Estimated glomerular filtration rate
CKD-EPI: Chronic Kidney Disease Epidemiology Collaboration
ECG: Electrocardiography
CT: Computed Tomography
SMD: Standardized mean difference
