# Supplementary material for: Prevalence and cardiovascular phenotypes of dextrocardia and situs inversus among 277,396 adults: longitudinal evidence of amplified age-related blood pressure progression
Source: Orphanet J Rare Dis. 2026 Jul 7;21:342. doi: 10.1186/s13023-026-04449-5 (PMC13637326; doi:10.1186/s13023-026-04449-5)
Supplement: Supplementary file 1 [file 13023_2026_4449_MOESM1_ESM.docx]

**Supplemental Material**

**Figure S1 Flowchart of participants included in the analyses of prevalence of dextrocardia and situs inversus.** Beijing's per capita life expectancy was 82.43 (≈83) years in 2020.

**Figure S2 Flowchart of participants included in the analyses of blood pressure differences in dextrocardia and situs inversus.** ‘N’ represents the number of records while ‘n’ indicates the number of participants. Outlier thresholds (0.025-99.975th percentiles) were determined using the distribution among participants without dextrocardia and situs inversus. Among 52 participants with dextrocardia or situs inversus, 8 had missing high-density lipoprotein cholesterol/low-density lipoprotein cholesterol values (imputed to preserve sample size) , while all other data met inclusion criteria without additional exclusions. Abbreviation: SBP, systolic blood pressure; DBP, diastolic blood pressure; BMI, body mass index; FBG, fasting blood glucose; eGFR, estimated glomerular filtration rate; TC, total cholesterol; TG, triglycerides; HDL, high-density lipoprotein cholesterol; LDL, low-density lipoprotein cholesterol; PP, pulse pressure.

**Figure S3 Flowchart of participants included in the analyses of long-term blood pressure changes in dextrocardia and situs inversus.** ‘N’ represents the number of records while ‘n’ indicates the number of participants. Among 88 records of 19 participants with dextrocardia or situs inversus, 6 records with missing value on low-density lipoprotein cholesterol (imputed to preserve sample size) , while all other data met inclusion criteria without additional exclusions. Abbreviation: SBP, systolic blood pressure; DBP, diastolic blood pressure; BMI, body mass index; FBG, fasting blood glucose; eGFR, estimated glomerular filtration rate; TC, total cholesterol; TG, triglycerides; HDL, high-density lipoprotein cholesterol; LDL, low-density lipoprotein cholesterol; PP, pulse pressure.

**Figure S4 Comparative smoothing curves of blood pressure versus age relationships using local polynomial regression model**. Results from 10% randomly selected participants versus the entire 227,834 participants without dextrocardia or situs inversus. The blue line represents the results from the 10% random subsample, while the red line shows the corresponding estimate from the entire 227,834 participants without dextrocardia or situs inversus.
